# Laparoscopic gynecological surgery under minimally invasive anesthesia: a prospective cohort study

**DOI:** 10.1007/s13304-022-01310-9

**Published:** 2022-06-27

**Authors:** Pierluigi Giampaolino, Luigi Della Corte, Antonio Mercorio, Dario Bruzzese, Antonio Coviello, Giovanna Grasso, Anna Claudia Del Piano, Giuseppe Bifulco

**Affiliations:** 1 Department of Public Health, School of Medicine, University of Naples Federico II, Naples, Italy; 2 Department of Neuroscience, Reproductive Sciences and Dentistry, School of Medicine, University of Naples Federico II, Naples, Italy; 3 Department of Anesthesiology and Intensive Care Medicine, Policlinico - Federico II University Hospital, Naples, Italy

**Keywords:** Gynecological surgery, Regional analgesia, Postoperative pain

## Abstract

The purpose of this study is to assess the feasibility and the perioperative outcomes of laparoscopic gynecological surgery in regional anesthesia (RA) from the point of view of the surgeon, anesthesiologist and patient. This is a prospective cohort study comprising sixty-six women planned to undergo gynecologic laparoscopy surgery for benign pathology at tertiary care gynecolgical center of the University Federico II of Naples. Women were assigned, according to their preference, to either RA (Group A) or general anesthesia (GA) (Group B). Surgical, anesthesiologic and postoperative recovery data were recorded. Postoperative pain was considered as the primary outcome. Secondary outcomes included mobilization, length of hospital stay, global surgeons and patient satisfaction, intraoperative pain assessment in Group A. Immediate postoperative pain was significantly lower in Group A 0 vs 2 (*p* < 0.001), with no significant differences at 24 h. The secondary outcome demonstrated early patient’s mobilization (*p* < 0.001) as well as early discharge (*p* < 0.001) and greater patient’s satisfaction for the Group A. In these patients, a maximum pain score of 3 points out of 5 was recorded through the entire surgery. RA showed to decrease the impact of surgical stress and to guarantee a quicker recovery without compromising surgical results. Although several surgical approaches can be employed to treat different conditions, RA technique could be a viable option for well-selected patients affected by gynecological diseases.

## Introduction

Outpatient surgery, defined as the surgical patient being admitted and discharged on the same day or within 24 h, is accountable for undoubted benefits such as satisfying patient preference to recover at home, lowering the risk of nosocomial infection, providing cost-effectiveness, and earlier mobilization [1, 2]. The pursuit of accomplishing “day surgery” is one of the main goals of any surgeon [3].

Laparoscopic procedures are commonly described as “minimally invasive” and the word minimal is attributed to surgical trauma, pain, hospitalization interval, scar [4].

Regional anesthesia (RA) from an anesthesiology perspective is the “minimally invasive technique” to achieve anesthesia. General anesthesia (GA) is the most common and used technique for laparoscopic procedures, however, it is responsible for different adverse effects in the postoperative period including the need for rescue analgesics, and antiemetics [5, 6]. Moreover, one of the main concerns observed in a patient scheduled for GA is preoperative anxiety. It can be generated for the fear of the unconscious state, losing control as well as the fear of awakening during the procedure. In addition, GA is in the collective imagination, synonymous with major surgical procedures and invasive high-risk surgeries [7, 8]. RA for the operative laparoscopic procedure has been largely applied for cholecystectomy procedures. It results in less surgical stress response, postoperative pain, lower incidence of postoperative nausea and vomiting, and rapid bowel canalization [9]. However, as regards, the gynecological domain, evidence about the outcomes of laparoscopy in RA are scarce. Trendelenburg procedure required for the gynecological procedure, worsening pulmonary compliance and generating discomfort for the patient, appears to be a great limit for the application of this technique [10]. Surgical gynecological procedures, under RA, are currently limited to diagnostic laparoscopy adnexectomy, ablation of endometriotic foci, and adhesiolysis [11, 12]. Only one case report on total laparoscopic hysterectomy is reported in the literature [13]. Recently, a review by Della Corte et al. has shown no significant advantages to using SA over GA for laparoscopic treatment of gynecological diseases [14].

The aim of our study was to assess the feasibility and the intraoperative and postoperative outcome of laparoscopic gynecological surgery under RA compared GA from the point of view of the surgeon, anesthesiologist and patient.

## Materials and methods

This is a prospective cohort study performed in a tertiary level referral center for minimally invasive gynecological surgery. All women who were referred to our center and met the inclusion criteria between January 2020 and April 2021 were enrolled.

Inclusion criteria were: women scheduled for laparoscopic surgery for benign gynecological conditions such as ovarian cyst (simple cyst, endometrioma, dermoid cyst) with a maximum diameter ≤ 8 cm, hydro/sactosalpinx, ectopic pregnancy (clinically stable conditions), primary/secondary infertility, patient carrier of BRCA mutation and aged more than 18 years old. The choice of including cysts ≤ 8 cm has been done to avoid cyst ruptures during manipulation of large ovaries and, so, the spread of possible neoplastic cells in the pelvis and abdomen. All women gave their written informed consent to participate in the study. Exclusion criteria were contraindications to GA o RA, including American Society of Anesthesiologists Physical Status Classification System (ASA score) IV, suspected malignancy, and BMI > 30 kg/m^2^,coagulopathy (acquired, induced, genetic), allergy to local anesthetics, patients with suspected malignancy, uncooperative patient for psychiatrics and neurological disorders (such as dementia and psychosis), increased intracranial pressure.

During the preoperative workup, all patients underwent gynecological examination and a detailed pelvic ultrasound scan was performed by an expert sonographer, eventually, MRI was performed to accurately define the characteristics of the lesion. Women were invited to participate in the study during the preoperative examination. After detailed and extensive counseling by the surgeon as well as by two anesthesiologists (AC and GG), with expertise about anesthesia in laparoscopic surgery and clinical and practical implication of both GA and RA, informed written consent was obtained and patient were allocated to one of the two groups according to their preferences.

Seventy patients were initially analyzed, but 66 patients satisfied the inclusion criteria and were enrolled in the study: 36 in Group A, 30 in Group B.

All the procedures were performed by a single operator (PG) with expertise in laparoscopic gynecological surgery who performed more than 100 procedures per year. The entire procedure was performed so that the patient could be invited to follow the entire procedure. A high-resolution color video screen was provided to show the intraoperative images. In group A, patients were informed about every single step of the intervention by both the surgeon and anesthesiologist. During each phase, patients were asked to score the pain using a Likert scale from 1 to 5.

Baseline demographic and clinical data of the patients included in the study as well as the intraoperative surgical and anestesiologic variables were recorded.

Postoperative pain assessed through Visual analog scale (VAS) was the primary outcome. The main secondary outcomes included: postoperative nausea and vomiting (PONV) and antiemetic/analgesic drugs usage. Further secondary outcomes were anesthesia complications, resumption of bowel motility, time to mobilization, global surgeons and patient satisfaction, length of hospital stay, intraoperative pain in RA group through Likert scale.

In the operating room, venous access was placed (18 G) and antibiotic prophylaxis was administered (Cefazolin 1 or 2 gr. iv, or in case of allergy, Clindamycin 600 mg iv) 30 min before skin incision, also dexamethasone 4 mg iv and midazolam 1 mg iv w administered. Vital signs were monitored: *Sp*O_2_, heart rate and blood pressure every 5 min.

In the sitting position, in group A, RA was performed at the T9-T10 or T10-T11 level. The level of puncture was confirmed by ultrasound counting the vertebrae from the sacrum, in a caudo-cranial sense. The technique was performed in asepsis. In the subarachnoid space after the vision of clear cerebrospinal fluid (CSF) in the spinal needle 27 Gauge, without letting out the CSF, Ropivacaine 0.375% 18 mg, Sufentanyl 7 mcg, and Clonidine 20 mcg were injected. Intraoperative sedation was carried out with midazolam 0.05 mg/kg and fentanyl 1 mcg/kg when pneumoperitoneum was performed. The anesthetic plane, suitable to the surgical procedure (T1-S4), was tested with the Pinprick and Ice test.

Group B patients undergoing GA received propofol (2 mg/kg), sufentanil (0.5 mcg/kg) and rocuronium bromide (0.6 mg/kg) for the induction of the anesthetic plane. The maintenance of the anesthetic plane was ensured with sevoflurane from 1 to 2%. Residual neuromuscular block antagonized with sugammadex 2–4 mg/kg about TOF. 

The management of postoperative pain was based on the administration of Paracetamol 1000 mg in the case of VAS < 5 and the administration of Ketorolac 30 mg in the case of VAS ≥ 5.

In case of inadequate analgesia, after 60 min of the Ketorolac after administration, Tramadol 100 mg i.v. was administered.

The incidence of PONV was considered and ondansetron 4 mg i.v. was administered in case of manifestation of the complication. If after 60 min PONV still occurred, dexamethasone 4 mg i.v. was administered. Pneumoperitoneum induction was achieved by open laparoscopy (Hasson technique) to avoid the high intraperitoneal pressure, otherwise necessary for the blind insertion of the first trocar, when performing the closed technique (Veress technique), and to prevent inferior epigastric artery damage [15]. Thus, the procedure was started with a low pressure of 8 mmHg and slowly increased to high flow, and pressure not higher than 11 mmHg was maintained throughout the entire surgery. Patients were placed into a minimal Trendelenburg position (maximum 20°) able to provide adequate visualization and bowel retraction. Ultrasound energy to cut and coagulate instead of monopolar/bipolar energy was used to perform salpingectomy or adnexectomy allowing to save time and reduce tissue trauma.

### Statistical analysis

All statistical analyses were conducted using the statistical Platform R (vers. 4.0.1).

Sample characteristics were reported using standard descriptive statistics. Mean ± standard deviation (min to max) in case of numerical variables and absolute frequencies and percentages in case of categorical factors. Numerical variables showing highly skewed distribution were described using median with interquartile range (25th–75th percentile). Accordingly, between-group comparisons were based on the *t*-test or the Mann–Whitney *U* test in case of numerical variables and the chi-square test or the Fisher exact test in case of categorical outcome. No correction for multiple comparisons was undertaken when analyzing longitudinal outcomes (VAS score and analgesic intake).

A sample size of 30 patients for group was deemed sufficient to highlight a large effect size (*d* = 0.8) with a power of 0.8 and a two-sided significance level of 0.05, using a Wilcoxon-Mann–Whitney *U* test on the primary outcome represented by the VAS score after 24 h from surgery.

## Results

All selected patients underwent surgical laparoscopy under regional (Group A) or general (Group B) anesthesia.

Baseline demographic and clinical data of the patients included in the study did not show significant differences between the two groups (Table 1).

**Table 1:** Patient’s characteristics

	Overall (*n* = 66)	Group A (*n* = 36; 54.5%)	Group B (*n* = 30; 45.5%)	*p* value
Age (years)	39 ± 16.1 (18–85)	39.9 ± 7.8 (27–52)	37.8 ± 22.4 (18–85)	0.625
BMI	23.7 ± 2.8 (19–30.5)	23.2 ± 2.4 (20.6–27)	24.3 ± 3.2 (19–30.5)	0.105
Comorbidity	23 (34.8)	13 (36.1)	10 (33.3)	1
Hypertension	5 (7.6)	1 (2.8)	4 (13.3)	
Thyroid disease	6 (9.1)	3 (8.3)	3 (10)	
Diabetes	3 (4.5)	3 (8.3)	0 (0)	
Other	9 (13.6)	6 (16.7)	3 (10)	
ASA				0.084
I	18 (27.3)	6 (16.7)	12 (40)	
II	42 (63.6)	27 (75)	15 (50)	
III	6 (9.1)	3 (8.3)	3 (10)	
Gynecological disease				0.003
Ovarian cyst	43 (65.2)	17 (47.2)	26 (86.7)	
Endometrioma	6 (9.1)	3 (8.3)	3 (10)	
Sactosalpinx	5 (7.6)	4 (11.1)	1 (3.3)	
Sterility	3 (4.5)	3 (8.3)	0 (0)	
Ectopic pregnancy	3 (4.5)	3 (8.3)	0 (0)	
BRCA mutation	6 (9.1)	6 (16.7)	0 (0)	
Trendelenburg’s position (degree)	16.5 ± 2.9	16.3 ± 3	16.7 ± 2.8	0.609
Operative Time (minutes)	61.3 ± 24.7	58.2 ± 24.8	65 ± 24.4	0.267

*BMI* Body Mass Index; *ASA* American Society of Anesthesiologists

Among the gynecological benign diseases, most of the patients had a simple ovarian cyst (43/66, 65.2%) in particular among Group B (26/30, 86.7%). No patient who underwent general anesthesia had sterility, ectopic pregnancy or BRCA1/2 mutation as surgical indication. Considering the type of intervention, although a statistically significant difference emerged between the two groups, this difference was not clinically relevant. No patients required laparotomic or anesthesia conversion and in no case accessory ports, in addition to the conventional four scheduled, were needed.

The degree of Trendelenburg’s position was similar in the groups: 16.3 ± 3 in Group A vs. 16.7 ± 2.8 degrees in Group B (*p* = 0.609). Also, operative time was not significantly different between the two groups (58.2 ± 24.8 in group A vs. 65 ± 24.4 min in group B, *p* = 0.267).

**Fig. 1:**
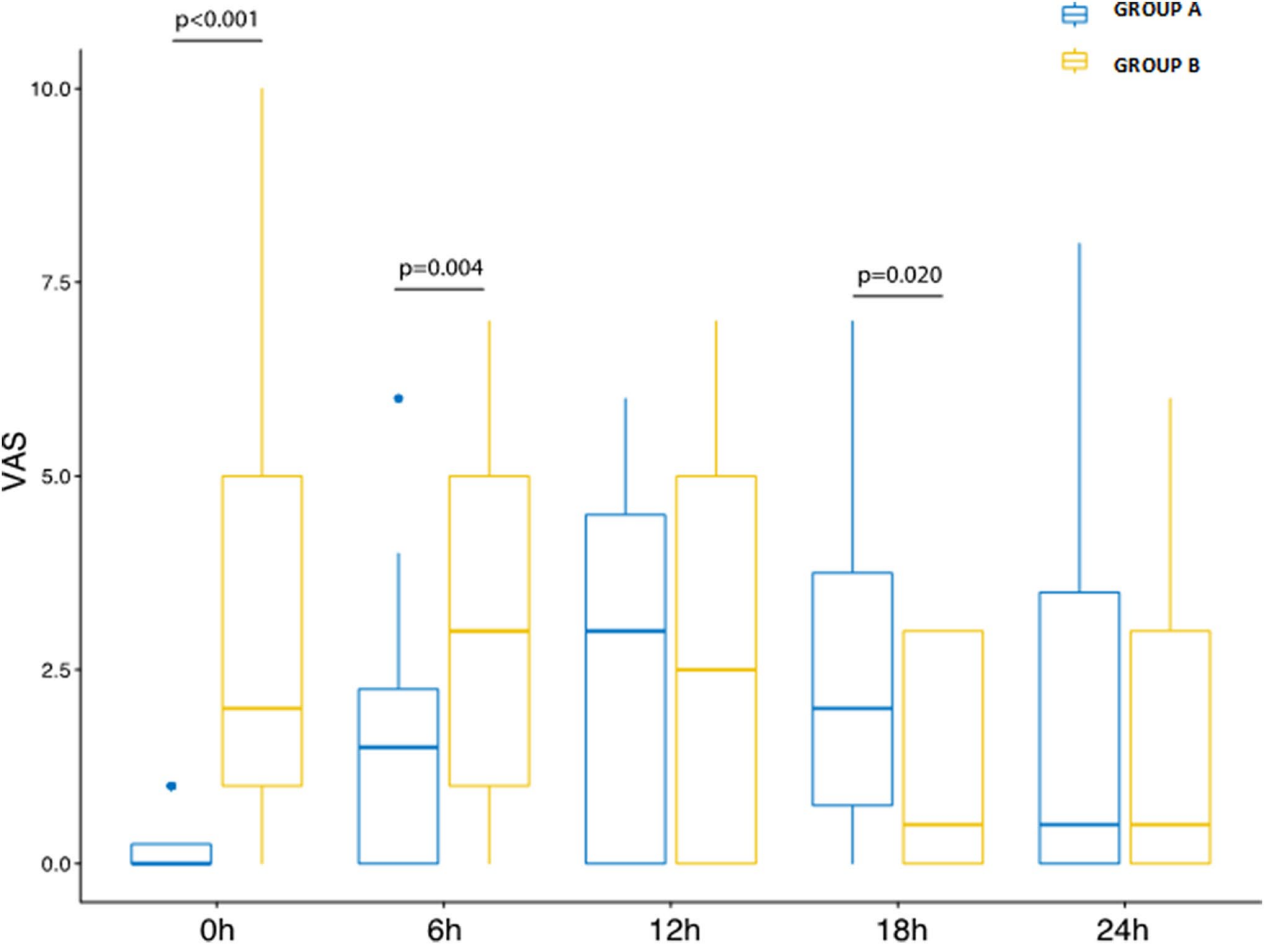
VAS score trend during the observation time in the two groups (0 to 24 h)

Regarding the postoperative pain (Table 2), the VAS score showed a change from 0 to 24 h (h): indeed, it was significantly lower in Group A up to 6 h [0 (0–0.8) vs 2 (1–5)], *p* < 0.001 in the immediate postoperative period and (1.5 (0–2.8) vs 3 (1–5), *p* 0.004 at 6 h) with an inversion at 18 h (2 (0.2–5.2) vs 0.5 (0–3), *p* 0.02) for Group B, and no statistically significant differences between the two groups at 24 h. Figure 1 reports the VAS score trend during the observation time (0–24 h). Analyzing the intake of analgesics after surgery, no differences were observed among the type of analgesic (paracetamol, ketorolac and tramadol) and during the post-operative observation time, except for paracetamol at 0 h, with a significant difference between the two groups (0 (0) vs 6 (20), (*p* = 0.007). In Table 3 are reported the secondary outcomes such as anesthesia related complications, resumption of bowel motility, patient’s mobilization, surgeons global satisfaction (the surgical team at the end of the procedure was asked about global satisfaction in term of pelvic organ exposure and ability to perform the procedure in relation to the anesthesia used), patient satisfaction (patients were asked to answer a closed-ended question upon discharge: would you do the same anesthesia again?), length in hospital stay. A faster resumption of bowel motility (7.1 ± 1.1 vs 13 ± 1.2, *p* < 0.001) and patient’s mobilization (2.9 ± 0.6 vs 6.7 ± 1.4, *p* < 0.001) and an early discharge (17.9 + 1.4 vs 23.8 ± 1.8, *p* < 0.001) were observed in Group A compared to Group B. No significant differences were recorded in term of PONV.

**Table 2:** Primary outcome

	Overall (*n* = 66)	Group A (*n* = 36; 54.5%)	Group B (*n* = 30; 45.5%)	*p* value
VAS score				
0 h	1 (0–1)	0 (0–0.8)	2 (1–5)	< 0.001
6 h	2 (0–4)	1.5 (0–2.8)	3 (1–5)	0.004
12 h	3 (0–5)	3 (0–5)	2.5 (0–5)	0.877
18 h	2 (0–3)	2 (0.2–5.2)	0.5 (0–3)	0.02
24 h	0.5 (0–3)	0.5 (0–4.5)	0.5 (0–3)	0.714
Analgesics intake				
Paracetamol 1 g				
0 h	6 (9.1)	0 (0)	6 (20)	0.007
6 h	9 (13.6)	3 (8.3)	6 (20)	0.28
12 h	12 (18.2)	6 (16.7)	6 (20)	0.758
18 h	9 (13.6)	3 (8.3)	6 (20)	0.28
24 h	12 (18.2)	6 (16.7)	6 (20)	0.758
Ketorolac 30 mg				
0 h	6 (9.1)	3 (8.3)	3 (10)	1
6 h	3 (4.5)	0 (0)	3 (10)	0.089
12 h	0 (0)	0 (0)	0 (0)	NA
18 h	0 (0)	0 (0)	0 (0)	NA
24 h	12 (18.2)	6 (16.7)	6 (20)	0.758
Tramadol 100 mg				
0 h	0 (0)	0 (0)	0 (0)	NA
6 h	0 (0)	0 (0)	0 (0)	NA
12 h	3 (4.5)	0 (0)	3 (10)	0.089
18 h	0 (0)	0 (0)	0 (0)	NA
24 h	0 (0)	0 (0)	0 (0)	NA

*VAS* Visual Analog Scale, *NA* not applicable

**Table 3:** Secondary outcomes

	Overall (*n* = 66)	Group A (*n* = 36; 54.5%)	Group B (*n* = 30; 45.5%)	*p* value
Anesthesia complications	3 (4.5)	2 (5.5)	1 (3.3)	0.245
PONV (no. (%))				
No	39 (59.1)	24 (66.7)	15 (50)	0.213
Yes	27 (40.1)	12 (33.3)	15 (50)	
Antiemetic drugs (No. patients)	9 (13.6)	3 (8.3)	6 (20)	0.280
Resumption of bowel motility (h)	9.8 ± 3.2 (5 to 15)	7.1 ± 1.1 (5 to 9)	13 ± 1.2 (11 to 15)	< 0.001
Length of hospital stay (h)	20.8 ± 3.4 (15 to 28)	17.9 ± 1.4 (15 to 21)	23.8 ± 1.8 (21 to 28)	< 0.001
Mobilization (h)	4.6 ± 2.2 (2 to 9)	2.9 ± 0.6 (2 to 4)	6.7 ± 1.4 (4 to 9)	< 0.001
Global surgeon satisfaction				0.136
Good	3 (4.5)	0 (0)	3 (10)	
Very good	12 (18.2)	6 (16.7)	6 (20)	
Excellent	51 (77.3)	30 (83.3)	21 (70)	
General patient satisfaction (would you do the same anesthesia again?)	63 (95.5)	36 (100)	27 (90)	0.089

Concerning anesthetic complications, we registered two cases of intraoperative hypotension (one for each group), managed with intravenous saline infusion, and only one case of bradycardia in group A. Two post-operative complications were recorded (according to Clavien-Dindo Classification I): one urinary tract infection (Group A) and one urinary retention (Group B) resolved with intermittent catheterization.

Finally, we reported for Group A the pain score obtained with the Likert scale, classically divided into 5 points (0: no pain, 5: maximum pain) during the various stages of surgery: introduction of uterine manipulator, introduction Hasson trocar and induction of pneumoperitoneum; introduction of ancillary trocars; exploration of pelvic organs; actual surgical procedure; skin suture. As reported in Table 4, all patients showed a pain score of 1 or 2, with only 3 cases (7.9%) with a score of 3 during the skin suture.

**Table 4:** Likert scale in Group A

Likert scale	Value (0–5)	Number of patients (%)
Introduction of uterine manipulator	1	33 (100%)
introduction of Verres needle	1	36 (100%)
introduction of Hasson and ancillary trocars	1	36 (100%)
induction of pneumoperitoneum	1 2	27 (75%) 9 (25%)
Exploration of pelvic organs	1 2	35 (92.1) 3 (7.9)
Actual surgical procedure	1 2	33 (84.6) 6 (15.4)
Skin suture	1/2 3	35 (92.1) 3 (7.9)

## Discussion

Our data confirm the suitability of laparoscopy surgery under RA for gynecological surgery, a still debated subject in literature, in contrast to general surgery of which successful results are already reported in literature [14–18].

Indeed, no need for laparotomic or general anesthesia conversion was required.

Based on our results, less pain was registered in RA group especially in the first postoperative hours. In addition, RA proved to lead to a quick recovery in terms of mobilization, resumption of bowel motility, length of hospital stays and was well accepted by both patients and surgeons.

Although we are fully aware that no other studies like randomized controlled trials can minimize bias and provide a rigorous tool to examine cause-effect relationships between intervention and outcome, our study was conceived and designed as a prospective study. We believe, indeed, that patient’s motivation and willingness to accomplish surgery under RA are key aspects for the accomplishment of the procedure therefore, in our opinion, randomization of patients at the moment has to be considered counterintuitive and challenging.

Strengths of our study were the sample size (the largest reported so far in literature), the accomplishment of all the procedures by one skilled surgeon and the assessment of the tolerability and the evaluation of the pain during each step of laparoscopy (to determine the acceptability of the procedure when performed under regional anesthesia). We consider this latter of paramount importance based on the assumption that sedation should be avoided as much as possible to preserve spontaneous breathing already restricted by the level of neuraxial anesthesia and Trendelenburg’s position.

An adequate and lasting post-operative pain control is crucial to enhance recovery [19]. Regarding postoperative pain, our result confirms the findings already registered in laparoscopy cholecystectomy under RA: less pain is registered especially in the first postoperative hour [16, 20, 21] and this can be considered of foremost importance as long as the early onset of pain right after surgery is capable to affect the whole recovery phase [19]^.^ This result might be explained by the persistent neuraxial blockade. To enhance this effect in our study clonidine was administered to the patient who underwent RA. Clonidine is an α2 adrenergic agonist used like an adjuvant in anesthesia. There were different possible mechanisms to explain the enhanced anesthetic efficiency. According to some researchers, the action of α2-agonism of clonidine induces vasoconstriction, which might contribute to prolonging the analgesia time. Furthermore, clonidine potentiates the spinal block via synergistic interaction between α2 receptors and sodium channels, resulting in a reduction in the dose of the local anesthetics required for achieving effective spinal anesthesia for certain surgical procedures [22, 23]^.^

Duration of surgery for women who underwent RA was comparable to the control group and the whole surgical team, questioned about pelvic organ exposure at the end of the procedure, gave in agreement positive feedback. These data, in particular, are encouraging given that one of the main concerns regarding the execution of this anesthesia for gynecological laparoscopic procedure is the difficulty to achieve a sufficient degree of Trendelenburg’s position (a key factor in gynecological surgery to retract bowel and provide adequate visualization) providing at the same time adequate ventilation. In our study, the degree of Trendelenburg’s position obtained in RA group was only 16.33 ± 2.97. No case of hypercapnia or pulmonary complication was registered.

RA compared with GA showed a quick recovery in the immediate postoperative setting. Enhanced Recovery After Surgery (ERAS) Protocols encourage the early mobilization of the patients [24]. Our data, according to the literature, demonstrate a significantly earlier mobilization in RA group compared to patient underwent general anesthesia. This finding together with the faster resumption of bowel movement, equally founded to be statistically significant in the RA group, is of particular interest in childbearing age women for the preventive effect on post-surgical adhesion formation (a proven factor for infertility disorders) [25]. GA has long been considered as causing a greater frequency and severity of PONV than regional anesthetic techniques [26]. In our study, patients in the RA group showed a lower incidence of PONV and required fewer drugs to avoid it, although these results were no statistically significant. Two previous studies evaluating PONV among gynecological patients treated with laparoscopy surgery with RA and GA showed discordant results. Raimondo et al. reported a higher incidence rate of PONV in women that received GA whereas Zirak et al. revealed this complication to be more frequent in the RA group [10, 27]. This divergence could be explained by the presence of a confounding factor, the inflation during laparoscopy, which can be considered by itself a cause of PONV [28, 29].

Although our study was not focused on cost analysis, based on our results we can globally consider RA a cost saving alternative to GA, in accordance with Turkstani et al. (who instead performed an accurate comparison of anesthesia cost about spinal versus general anesthesia for laparoscopic cholecystectomy) [30], taking in consideration the reduced amount of drugs used for our patients in the postoperative time and the earlier discharge.

Great motivation for the patient is mandatory to accomplish this technique and patient anxiety must be addressed before surgery with appropriate counseling but communication with an empathetic anesthesiologist and surgeon just during surgery may help significantly to reduce patients' anxiety. In this regard, we considered it essential to inform the patient about each step of the surgery (showing on request the live procedure on the screen) and obtain his feedback even during surgery.

Considering all the steps, a maximum of 2 points on the Likert scale (considered a mild pain) was recorded: only 3 cases with a score of 3 during skin suture were registered.

Finally, another important advantage of RA, to not be underestimated during the ongoing COVID-19 pandemic, is the avoidance of airway management that can prevent the risk of virus spread [31].

## Conclusions

Based on the aforementioned results we consider RA a valid alternative to GA for laparoscopic gynecological surgery: in accordance with the goals of the minimally invasive surgery era, RA demonstrated to decrease the impact of surgical stress and to guarantee a quicker recovery without compromising, at the same time, surgical outcomes. As well as different surgical approaches could be selected based on patients characteristics and diseases, accordingly, anesthesia’s technique should be tailored on patients motivations and conditions. However, further studies are required to evaluate more complex and longer surgical procedures.

## Data Availability

The datasets generated during the study are available from the corresponding author on reasonable request.
